# Rotating cluster mechanism for coordinated heterogeneous MIMO cellular networks

**DOI:** 10.1186/s13638-018-1061-1

**Published:** 2018-03-14

**Authors:** Hakimeh Purmehdi, Robert C. Elliott, Witold A. Krzymień, Jordan Melzer

**Affiliations:** 1 Department of Electrical and Computer Engineering, University of Alberta, T6G 1H9 Edmonton, Alberta, Canada; 2 TELUS Communications, Ottawa, Ontario Canada

**Keywords:** Heterogeneous multiple-antenna cellular networks, Multiuser MIMO downlink, Network coordination, Cell clustering, Inter-cell and inter-cluster interference mitigation, Cluster pattern rotation

## Abstract

To increase the average achievable rates per user for cluster-edge users, a rotating clustering scheme for the downlink of a coordinated multicell multiuser multiple-input multiple-output system is proposed in this paper and analyzed in two network layouts. In the multicell heterogeneous cellular network, base stations of a cluster cooperate to transmit data signals to the users within the cluster; rotating cluster patterns enable all users to be nearer the cluster center in at least one of the patterns. Considering cellular layouts with three or six macrocells per site, different rotating patterns of clusters are proposed and the system performance with the proposed sets of clustering patterns is investigated using a simulated annealing algorithm for user scheduling and successive zero-forcing dirty paper coding as the precoding method. The rotating clustering scheme is less complex than fully dynamic clustering, and it is primarily designed to improve the throughput of cluster-edge users. As an extra secondary benefit, it is also capable of slightly improving the average achievable sum rate of the network overall. The effectiveness of the proposed methods with two different scheduling metrics, namely throughput maximization and proportionally fair scheduling, is of interest in this work. Moreover, the speed of rotation affects the performance of the system; the higher the speed of rotation, the more frequently any specific users will be nearer the cluster center. Our simulations demonstrate the effectiveness of the proposed rotational approach and determine the speed of rotation beyond which any additional performance gains become negligible.

## Introduction

Exploding demand for ever-higher data throughputs in cellular networks is one of the main drivers behind their current evolution toward the 5th generation [1]. As a promising solution for increased throughput, multiuser multiple-input multiple-output (MU-MIMO) techniques have been introduced [2], in which several single- and/or multiple-antenna users receive their corresponding signals simultaneously from multiple-antenna base stations (BSs). High spectral efficiency gains potentially achievable with MIMO spatial multiplexing are available at high signal-to-interference-plus-noise ratios (SINRs). However, to maximize capacity, cellular networks are normally designed to allow high levels of inter-cell interference, which prevents high spatial multiplexing gains. Network coordination (also known as coordinated multipoint (CoMP) transmission/reception or network MIMO) is one potential solution to reduce the inter-cell interference [3–6].

Historically, the key driver behind dramatic increases in area capacity of cellular networks has been reduction of cell sizes and densification of cellular layouts. More recently, this trend has evolved into the development of dense heterogeneous networks (HetNets) [7–9]. Coordinated transmission on the downlink of MU-MIMO HetNets is considered in this work. Since coordination of all BSs in a large cellular network is neither practical nor necessary, coordination of transmissions within limited-size clusters of BSs is considered instead [10, 11]. Although the SINR of most users is improved by coordination of clustered BSs, the inter-cell interference is now replaced by inter-cluster interference (ICI). Users located near the edge of the cluster experience much higher levels of ICI than users closer to its center, and they will suffer from poor throughput or even service starvation. A proportionally fair (PF) user scheduler [12–15] will improve the throughput and fairness to these users. To improve the performance of cluster-edge users, clusters can change and be formed dynamically [16–21]. This increases the possibility of any given user being near the center of a cluster for at least a portion of the time.

Clusters can be formed in different ways. In the simplest method, i.e., using a fixed pattern, BSs of the cluster are assigned with a predefined pattern that remains static during the system operation [3, 22]. This method has comparatively low complexity and overhead, but cluster-edge users still adversely suffer from ICI. In fully dynamic clustering, all features of the cluster such as the size and/or shape of the clusters and the set of BSs forming a cluster can be changed as often as every scheduling interval [16–18, 21, 23–30]. Clustering can be controlled and managed with a centralized processor using channel state information (CSI) of involved users [16, 21, 23–30]. This method is quite complex, but it is able to improve the performance of the system considerably in comparison to that of a fixed pattern. For example, [30] introduced a type of dynamic clustering algorithm based on weighted sum rate maximization for the downlink of a MU-MIMO CoMP system. The clusters were formed specifically for the set of scheduled users such that they experience minimum ICI. It was shown that the algorithm improved the system sum rate in comparison to that of static clustering methods.

A simplified version of dynamic clustering is cluster rotation, in which the complexity and overhead burden on the centralized processor are reduced by using one of several predefined patterns of clustering that change periodically. Thus, the cluster-edge effect is averaged out and diminished over time since users, who are periodically located either near the border or the center of the clusters as the cluster borders change with each pattern, each receive data primarily during their most favorable pattern(s). In [19], we proposed a method of cluster rotation for the downlink of a multicell MU-MIMO HetNet, and its performance was compared with static clustering using both maximum throughput (MT) and PF scheduling metrics. A simulated annealing user scheduling algorithm and successive zero-forcing dirty paper coding (SZF-DPC) [31] precoding were used in the system. We demonstrated that instantaneous achievable rates of users and the average achievable per-user rates were improved for the lowest throughput users (i.e., cluster-edge users). Also, the average achievable sum rate was improved compared to fixed clustering, especially for PF scheduling. Naturally, enforcing fairness in the system reduced average per-user rates for the highest throughput users under MT scheduling. We also evaluated the effect of different cluster rotation speeds on the system performance under PF scheduling in [20]. The results demonstrated that faster rotation in general performed better than slow rotation. However, there was an upper limit on increasing the rotation speed, and beyond that point, further increases did not result in any notable additional gains in sum rate or per-user rate.

The macro BSs in [19, 20] were each equipped with one out of six sets (per site) of antenna arrays, with each set covering a cell; the hexagonal-shaped macro site coverage area thus was divided into six cells^1^. We are interested to know whether the results in [19, 20] can be generalized into other types of network layouts and/or larger sets of possible cluster patterns, and how the rotating cluster patterns can be modified for those other layouts. This generalization aspect is quite important; cluster rotation would not be nearly so useful, if it only worked for a certain type of network layout. Thus, we extend our rotating clusters idea to a layout with three macrocells per site and attempt to find a suitable set of rotating patterns that can improve the performance of the system. The clover-leaf-shaped cell layout is the most suitable three-cell layout to use, and it is recommended by ITU-R for IMT-Advanced [32, 33]. Because of the closer resemblance between the cellular contour and the coverage of the clover-leaf-shaped layout [34], it is also more often used in modeling practical cellular systems than the simple hexagonal layout. Assuming a clover-leaf-shaped cell layout, we propose a set of five rotating cluster patterns and again compare the system’s performance in terms of the achievable throughput with that of static clusters under both MT and PF scheduling metrics. It should be noted that while we examine only two regular grid-like network layouts in this work, the concept of cluster rotation can be also applied to more general, irregular layouts.

To keep the same general methodology as in [19], we continue to use SZF-DPC precoding, which partially nulls the interference between users [31]. Since reduced-complexity user scheduling is necessary in practical systems with a large number of users, we use our previously proposed simulated annealing user scheduling (SAS) method [35]. It was shown in [35] that this method achieves performance close to that of an exhaustive search with much lower complexity, making its continued use in this context reasonable. The SAS algorithm in [19] was memoryless, while in [20], we added memory to the algorithm to further improve its performance. We have made additional refinements to the SAS algorithm in [35]; it is that version (adapted for weighted sum rates) that we use in this work. We wish to emphasize, though, that the focus of this work is on the cluster rotation and its performance, not on the scheduling algorithm, nor on the precoding method. The cluster rotation methods employed in this work can be generalized and applied both to different user scheduling algorithms and different precoding methods, including (but not limited to) those discussed in [35]. The outcomes of this work are expected to be independent of the user scheduling and/or precoding approaches. Please recall that the cluster rotation scheme addresses the impact of *inter*-cluster interference, while precoding and user scheduling attempt to resolve the *intra*-cluster interference. Hence, SZF-DPC precoding and the SAS algorithm are simply meant to serve as a representative case.

Additionally, compared to [19, 20], we have revised some of the simulation parameters in this paper to correspond to recommendations in [32, 33]. These include the values of the path loss exponent and the standard deviation of shadow fading for macro and pico BSs, along with their transmitted power levels. We also have corrected a minor flaw in our simulations from [19]. There, when two adjacent 60° cells are coordinated, they have erroneously been given a single 120° antenna pattern. We have corrected this misbehavior so the coordinated cells now indeed have two 60° patterns as intended. (This misbehavior was also corrected in [20].) Lastly, we provide a comparison of the performance of cluster rotation with that of fully dynamic clustering, specifically the scheme described in [30].

The notation used in this paper is as follows. An italic variable *a* or *A* denotes a scalar, while boldface lowercase and uppercase variables **a** and **A** denote a vector and matrix, respectively. **I**_*M*_ is the *M*×*M* identity matrix. **A**^*T*^ and **A**^*H*^ denote the transpose and conjugate (Hermitian) transpose, respectively, of **A**. For a square matrix **A**,*Tr*(**A**),**A**^−1^, and **A**^−1/2^ are its trace, inverse, and inverse square root, respectively. **A**≽**0** denotes **A** is positive semi-definite. |*a*| denotes the absolute value of the variable, and |**A**| denotes the determinant of a (square) matrix.

## System model, design, and achievable weighted sum rate maximization

We consider the downlink of a coordinated multicell MU-MIMO HetNet. Several macro BSs are co-located at each macro site, the coverage of which is partitioned into different cells each covered by an antenna array installed on a macro BS. We assume two different network layouts, the first with six macrocells per site, and the second with three macrocells per site. (For shorthand, we refer to these respectively as “six-cell” and “three-cell” layouts in this paper.) Different system model characteristics are assumed, which are described in Sections 2.1 and 2.2, respectively, for the six-cell and three-cell layouts. In both system models, omnidirectional pico BSs surround each macro site and overlay the macro coverage area. The macro BSs each transmit with power *P*_*t*_, and the inter-site distance (ISD) between macro sites is fixed and denoted by *D*. Each macro BS is equipped with *N*_macro_ transmit antennas, while each pico BS has *N*_pico_ antennas. We assume for simplicity that *N*_macro_ and *N*_pico_ are both equal to *N*. Since all macro and pico BSs in a cluster jointly transmit their signals, essentially virtually forming one large antenna array, this assumption is reasonable and does not impact the proposed methods or the analysis in this work. Having *N*_macro_ larger than *N*_pico_ would of course increase the system sum rates and the number of users that could be scheduled simultaneously, but would also thereby significantly (and likely needlessly) increase the complexity and length of time of relevant simulations. Additionally, the examination herein assumes perfect and instantaneous CSI and data shared across the backhaul of the network. A centralized processor for the network is assumed to collect the CSI, e.g., by (idealized) feedback from the mobile users of the CSI obtained using pilot reference signals; it also performs scheduling and coordinates the transmissions between BSs. The specifics of the CSI gathering and coordination over the backhaul are outside the scope of this paper.

### Layout 1: six-cell layout (hexagonal-shaped cooperating area)

**Fig. 1:**
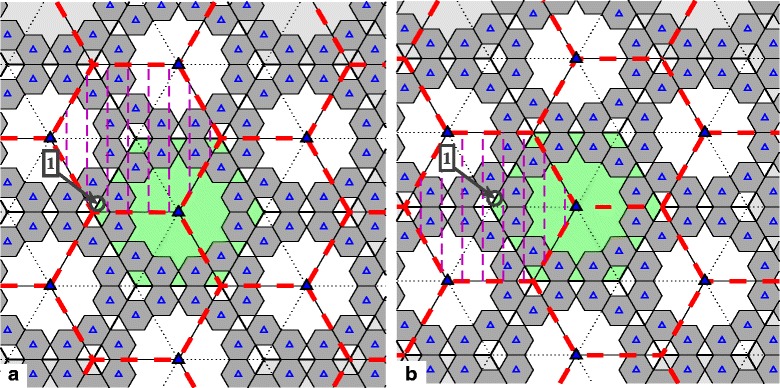
Network layout with six cells per macro site for HetNet with cluster rotation: **a** and **b** depict two alternating clustering patterns of BS coordination. Solid and open triangles represent macro BS sites and pico BSs, respectively, and the thick red dashed hexagons denote clusters

In our first HetNet model, the coverage area of the six cells per site overall forms a hexagonal-shaped region. Each macro BS covers a 60° angle of the area with a directional antenna. The macro site is surrounded by 12 low-powered pico BSs that form picocells overlaying the macro coverage area (see Fig. 1). Of the six cells per site, two adjacent ones are coordinated at any given time to form an effectively larger cell area. The picocells also coordinate within whatever cluster that the macrocell they overlay is part of. Without loss of generality, we may consider any arbitrary macro site (with coverage area shown in green) and the clusters it participates in (shown by the red dashed lines). Therefore, the BSs of any macro site contribute to three different clusters.

As depicted in Fig. 1, two different patterns of clustering are possible, in which different adjacent cells cooperate with each other. All cells within each thick red dashed hexagon coordinate signals from their BSs to form a cluster; one example cluster in each pattern is emphasized in the figure for clarity. As Fig. 1a depicts, those users in a cluster that are located near the border of the cluster (for example, at location “1”) experience the poorest channel conditions from the BSs in the cooperating set. By rotating the clustering pattern by 60° around any macro site (see Fig. 1b), those previously poor-coverage users are now in the middle of the cluster (i.e., they will have better channel gains or higher achievable rates). Therefore, most users will have the opportunity to have a higher chance of being scheduled and to achieve reasonably good data rates for a fraction of the overall transmission time. With users being scheduled primarily during their most favorable clustering pattern, their corresponding rates will be higher than otherwise. Averaging the throughput over all transmission periods and clustering patterns, the overall achievable transmission sum rate of the users will be improved.

There are *K* users uniformly distributed over the coverage area of each macro site, each user equipped with *M* receive antennas. *K*_*c*(*i*)_ is the number of users assigned to cluster *c*(*i*), from which *U*_*c*(*i*)_ users are served, where *i* refers to the *i*th pattern of clustering. Each cluster transmits coordinated data signals from all its BSs to its scheduled users.

### Layout 2: three-cell layout (clover-leaf-shaped cooperating area)

**Fig. 2:**
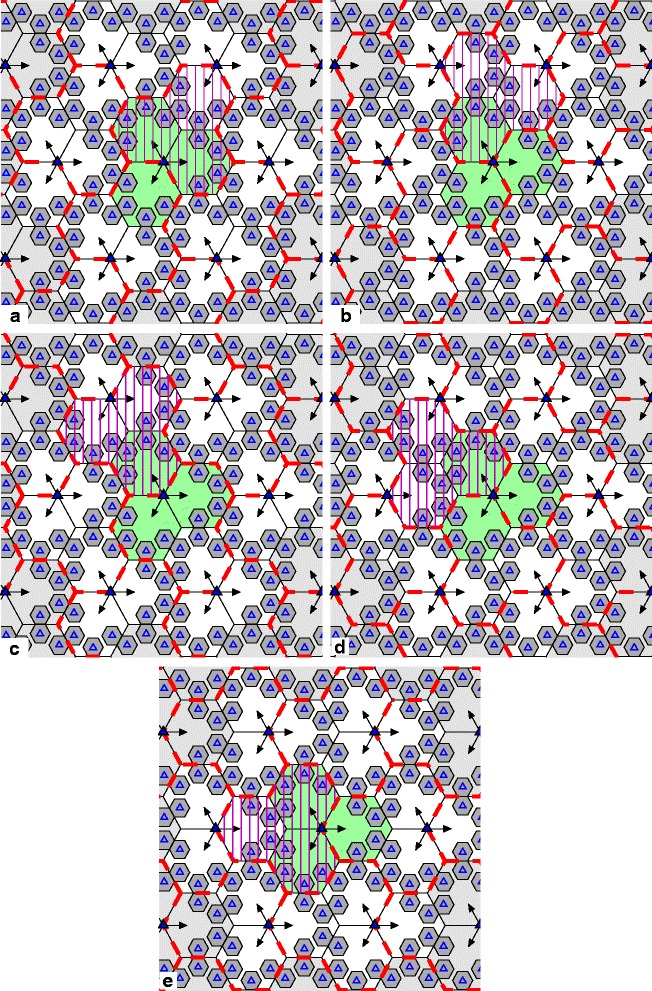
Network layout with three cells per macro site for HetNet with cluster rotation: **a**–**e** depict five different clustering patterns of BS coordination. Solid and open triangles represent macro BS sites and pico BSs, respectively, and thick red dashed lines denote cluster borders

For the second HetNet model, which is more commonly used in LTE-advanced design [32] and is called a clover-leaf model, each cell in a macro site is covered by a high-powered BS, which is located at a corner of the cell. The directional antenna at a macro BS covers a hexagonal-shaped cell within the angle of 120°. Each macrocell is overlaid by four low-powered omnidirectional pico BSs. These are located near the four edges of the macrocell that are the most distant from the macro BS, as depicted in Fig. 2. Any three adjacent macrocells and their constituent picocells may form a cluster, if the macrocells share a corner that is not a site. Therefore, considering an arbitrary macro site and its corresponding three macrocells (shown in green in Fig. 2), the macro BSs may belong to two or three independent clusters (shown by the red dashed lines).

As depicted in Fig. 2, five different patterns of clustering are possible. We again highlight one example cluster in each pattern for clarity. Those users that are located near the edge of the cluster experience poor channel conditions from the BSs in cooperating set. Consequently, their achievable rates will be smaller compared to the users in the middle of the cluster. By rotating the clustering pattern (see Fig. 2b), a portion of those previously poor-coverage users are now in the middle of the cluster, and some of the users, previously located at the middle of cluster, are now near the edge of the cluster. To put the remainder of the cluster-edge users in Fig. 2a near the middle of a cluster, more rotations are required, which are depicted in Fig. 2c–e. Therefore, in this layout after five intervals of rotation all users have the opportunity of being at least once in the middle of the cooperating area; they thus have a higher chance of being scheduled and achieving reasonably good data rates.

There are *K* users, each equipped with *M* receive antennas, uniformly distributed over each cell. This is in contrast to the six-cell layout, which has *K* users distributed over the coverage area of the macro site. Thus, for the three-cell layout, there are 3*K* users per site, i.e., *K*_*c*(*i*)_=3*K*.

For both layouts, other patterns of clustering could in theory be used, e.g., by coordinating more macrocells together in a cluster. However, please consider the corners of each macrocell that do not contain a macro BS site. These locations have the worst SINR when no coordination occurs. The patterns that we use cluster the smallest possible number of macro BSs such that it allows each of those corners to be in the center of the cluster in one of the patterns. At the same time, the duration between any given corner being in the center (as the scheme rotates through the patterns) is also the smallest possible for the number of macrocells per cluster being used. (Note there are two of these corners per macrocell in the six-cell layout, and five such corners in the three-cell layout, hence leading to two and five patterns, respectively, for the layouts.)

### Cluster rotation in general network layouts

There are, in general, two “rotation” aspects to cluster rotation. The first can be viewed as a physical rotation. Please note the highlighted cluster in Figs. 1 and 2 (denoted by dashed and solid vertical lines, respectively). As the cluster patterns change, that cluster, in a sense, can be imagined as rotating around some location in the network. In the two cases depicted by Figs. 1 and 2, that location is the macro site in the middle of each subfigure, though this need not be the case in general. The second aspect of rotation is the periodic rotation through a set of cluster patterns, in a round-robin fashion. This latter aspect is more general to any arbitrary cell layout. The first aspect may not necessarily be applicable, or at least quite so readily visible, as the second. For example, the five patterns in Fig. 2 could be ordered arbitrarily. If so, the physical rotation aspect would not be as apparent, but the rotation through the set of (re-ordered) patterns would still occur.

While we investigate two regular grid-like cell layouts herein, the concept of cluster rotation can also be applied to more general irregular layouts. For such irregular layouts, it would first be necessary to determine sets of BSs in the network for coordination and then assign different clustering patterns to them. This may not be as simple as with a regular cell layout, but remains feasible, given a set of BS locations and coverage areas and/or where interference results without coordination. Voronoi diagrams of order *n* [36] could be of use to locate regions of coordinated BSs, by identifying the *n* nearest BSs at any given location; the distances should also be weighted based on the type/tier of each transmitting node. The system can then rotate through those patterns just as in this work.

### Complexity comparison of dynamic clustering and rotating clustering

Fully dynamic clustering (whether the scheme in [30] or otherwise) results in significantly higher overhead in computational load and signaling. The system must determine and exchange possible choices of BSs for each user, run some sort of optimization or other routine to determine the choice of which BSs to serve which user, and finally communicate these choices across the network and to the users. This could occur potentially as often as every scheduling interval, though the system could also perform these operations less frequently. In comparison, almost none of those computations are required with rotating clustering since the sets of clusters are predetermined beforehand and known at all transmitting nodes. The additional overhead beyond that of static clustering is simply the same as the last stage of fully dynamic clustering, i.e., to periodically inform the users what their new cluster will be.

Furthermore, there are additional savings in complexity in regard to cell association. With rotating clustering, the association of a user to a specific anchor BS has much less impact on the network’s operation (disregarding the context of high user mobility and/or handoff, which are outside the scope of this work). Note that a user receives data from a macrocell and all picocells overlaying that macrocell. Borders between macrocells (where the received power from the BSs of those cells are equal) are statistically identical; at times, that cell border may also be a cluster border, while at other times, it will not. Thus, a complicated cell association scheme is not required. Whether a user chooses an anchor BS by closest distance, highest average received power, adding on a tier-dependent association bias factor, etc., the performance of the scheme is by and large unchanged. Essentially, users can be considered more to be associated with a cluster rather than with an individual cell; in terms of performance, it is largely equivalent to associate with any one of the cells in the cluster. There may still be, for example, considerations of offloading traffic, but these would now be between clusters rather than between cells. In any event, such factors are beyond the scope of this paper.

### Achievable weighted sum rate maximization and user scheduling

For both layouts, as stated earlier, averaging the throughput over all transmission periods and clustering patterns will improve the overall achievable sum rate of the users, with users scheduled primarily during their most favorable pattern. Defining *T*_*cl*_ as a specific clustering pattern duration in units of scheduling intervals, rotation to the next pattern will occur every *T*_*cl*_ scheduling intervals. Denoting *B*_*c*(*i*)_ as the number of BSs in the *c*(*i*)th cluster of the *i*th pattern, the aggregate downlink channel $\mathbf {H}_{c(i),k} \in \mathcal {C}^{M\times B_{c(i)} N}$ of the *k*th user from all these *B*_*c*(*i*)_ BSs is defined by **H**_*c*(*i*),*k*_=[**H**_*c*(*i*),*k*_(1),⋯,**H**_*c*(*i*),*k*_(*B*_*c*(*i*)_)], where $\mathbf {H}_{c(i),k} (b) \in \mathcal {C}^{M\times N}$ denotes the downlink channel matrix between the *k*th user and *b*th BS of the cluster. Each element of **H**_*c*(*i*),*k*_(*b*), denoted by *h*_*c*(*i*),*k*_(*b*,*m*,*n*), is the complex downlink channel signal strength coefficient between the *m*th receiving antenna of the *k*th user and the *n*th transmitting antenna of the *b*th BS in the *c*(*i*)th cluster. This coefficient includes path loss, log-normal shadowing, and Rayleigh fading, and is modeled by

1 $$ {\begin{aligned} h_{c(i),k} (b,m,n)&=z_{c(i),k} (b,m,n)\\ &\quad\times \sqrt{\Gamma_{0} P_{t} (b)\! \left(\frac{R_{m}}{d_{c(i),k} (b)}\right)^{\alpha (b)} \! \rho_{c(i),k} (b)A(\theta,\! b)}. \end{aligned}}  $$

*z*_*c*(*i*),*k*_(*b*,*m*,*n*) represents small-scale frequency-flat Rayleigh fading with an i.i.d. complex Gaussian random variable distributed as $~\mathcal {CN}(0,1)$. *R*_*m*_ is the reference distance^2^, and *Γ*_0_ is a scaling factor controlling the reference signal-to-noise ratio (SNR) at a distance of *R*_*m*_ in the boresight direction of the directional antenna. The distance between user *k* and BS *b* in cluster *c*(*i*) is represented by *d*_*c*(*i*),*k*_(*b*), and *α*(*b*) is the path loss exponent for BS *b*. *P*_*t*_(*b*) is the transmit power of BS *b*, and *ρ*_*c*,*k*_(*b*) denotes the log-normal shadow fading coefficient with standard deviation *σ*_*ρ*_. The antenna pattern *A*(*θ*,*b*) of a macro BS, where *θ* is the angle between the direction of interest and the boresight of the antenna at BS *b*, is defined as described in [32, 37]; *A*(*θ*,*b*) is equal to unity for pico BSs with omnidirectional antennas.

All *B*_*c*(*i*)_ BSs of cluster *c*(*i*) cooperatively transmit the data vector $\mathbf {s}_{c(i),k} \in \mathcal {C}^{M\times 1}$ for user *k* using the aggregate precoding matrix $\mathbf {W}_{c(i),k} \in \mathcal {C}^{B_{c(i)} N\times M}$. The received signal $\mathbf {y}_{k} \in \mathcal {C}^{M\times 1}$ for user *k* is given by

2 $$ {{\begin{aligned} {}\mathbf{y}_{k} = \mathbf{H}_{c(i),k}\sum_{j=1}^{U_{c(i)}}\mathbf{W}_{c(i),j}\mathbf{s}_{c(i),j} +\! \underbrace{\sum_{\check{c}(i)\neq c(i)}\mathbf{H}_{\check{c}(i),k}\sum_{\forall j}\mathbf{W}_{\check{c}(i),j}\mathbf{s}_{\check{c}(i),j}+\mathbf{n}_{k}}_{\mathbf{Z}_{c(i),k}}. \end{aligned}}}  $$

The first term in (2) is the received signal from cluster *c*(*i*), to which the user belongs, while the second term describes the interference from other clusters. Applying the central limit theorem, the total interference signal from all clusters not including *c*(*i*), denoted by $\check {c}(i)\neq c(i)$, is approximated by an *M*×1 complex Gaussian random vector with zero mean and standard deviation *σ*_*I*_. To estimate the standard deviation of this interference, it is assumed that all BSs outside the cluster *c*(*i*) are transmitting with full power, representing the worst case for ICI. The interference from these BSs experienced at different locations within the cluster *c*(*i*) is determined and averaged via Monte Carlo simulation over many channel realizations. The standard deviation of these realizations is used as the value of *σ*_*I*_. The last term $\mathbf {n}_{k} \in \mathcal {C}^{M\times 1}$ is a complex additive white Gaussian noise vector with each element having zero mean and unity variance. The summation of interference and noise is denoted by **Z**_*c*(*i*),*k*_, which with the Gaussian interference approximation ends up as a complex Gaussian random vector with zero mean and variance $\sigma _{I}^{2} + 1$. For convenience of calculation, the interference-plus-noise power is normalized at the receiver. This is equivalent to applying a filter at the receiver of $\mathbf {Q}_{r} = \left (\sigma _{I}^{2} + 1\right)^{-1/2}\mathbf {I}_{M}$. Hence, by defining $\tilde {\mathbf {H}}_{c(i),k}= \mathbf {Q}_{r}\mathbf {H}_{c(i),k}$ as the post-processed equivalent channel matrix and $\tilde {\mathbf {Z}}_{c(i),k}= \mathbf {Q}_{r}\mathbf {Z}_{c(i),k}$ as the normalized interference plus noise, (2) is revised as

3 $$ \tilde{\mathbf{y}}_{k} = \tilde{\mathbf{H}}_{c(i),k}\sum_{j=1}^{U_{c(i)}}\mathbf{W}_{c(i),j}\mathbf{s}_{c(i),j} + \tilde{\mathbf{Z}}_{c(i),k}.  $$

We choose to use the SZF-DPC precoding technique, where the encoding order of the users is very important for maximization of the achievable weighted sum rate. Given a set of users with order $\pi _{c(i)}^{j}$ and defining the user encoded at position *k* as $\pi _{c(i),k}^{j}$, the post-processed received signal can be modified and expanded as

4 $$  \begin{aligned} \tilde{\mathbf{y}}_{\pi_{c(i),k}^{j}} & = \tilde{\mathbf{H}}_{c(i),\pi_{c(i),k}^{j}}\mathbf{W}_{c(i),\pi_{c(i),k}^{j}}\mathbf{s}_{c(i),\pi_{c(i),k}^{j}} \\ & \quad+ \tilde{\mathbf{H}}_{c(i),\pi_{c(i),k}^{j}}\sum\limits_{l<k}\mathbf{W}_{c(i),\pi_{c(i),l}^{j}}\mathbf{s}_{c(i),\pi_{c(i),l}^{j}} \\ & \quad+ \tilde{\mathbf{H}}_{c(i),\pi_{c(i),k}^{j}}\sum\limits_{l>k}\mathbf{W}_{c(i),\pi_{c(i),l}^{j}}\mathbf{s}_{c(i),\pi_{c(i),l}^{j}} \\ & \quad+ \tilde{\mathbf{Z}}_{c(i),\pi_{c(i),k}^{j}}. \end{aligned}  $$

The two summations in the second and third line of (4) represent the intra-cluster interference for user *k*. In SZF-DPC, the precoding matrix $\mathbf {W}_{c(i),\pi _{k}^{j} }$ is constrained to lie in the null space of the channel matrices of all users encoded before $\pi _{c(i),k}^{j}$; the aggregate channel matrix of previously encoded users is defined as $\mathbf {H}_{k-1}=\left [\tilde {\mathbf {H}}_{c(i),\pi _{c(i),1}^{j}}^{T},\ldots,\tilde {\mathbf {H}}_{c(i),\pi _{c(i),k-1}^{j}}^{T} \right ]^{T}$. The precoding matrix cancels the intra-cell interference from the summation in the third line of (4), while the effect of the remaining intra-cell interference represented by the summation in the second line of (4) is removed by using DPC. Using singular value decomposition of **H**_*k*−1_, for a given ordered user $\pi _{c(i),k}^{j}$, its achievable rate $R_{c(i),\pi _{c(i),k}^{j}}$ is given by

5 $$ \begin{aligned} {} R_{c(i),\pi_{c(i),k}^{j}} &= \log_{2}\left|\mathbf{I}_{M} + \left(\tilde{\mathbf{H}}_{c(i),\pi_{c(i),k}^{j}}\mathbf{V}_{k-1}^{0}\right) \right.\\ &\qquad \qquad\quad \times \left. \mathbf{Q}_{c(i),\pi_{c(i),k}^{j}}(\tilde{\mathbf{H}}_{c(i),\pi_{c(i),k}^{j}}\mathbf{V}_{k-1}^{0})^{H}\right|. \end{aligned}  $$

$\mathbf {Q}_{c(i),\pi _{c(i),k}^{j}}$ is the transmit covariance matrix for user $\pi _{c(i),k}^{j}$ in cluster *c*(*i*), and $\mathbf {V}_{k-1}^{0}$ are orthonormal basis vectors for the joint null space of **H**_*k*−1_ for the users before $\pi _{c(i),k}^{j}$ in the encoding order; $\mathbf {V}_{0}^{0} \triangleq \mathbf {I}_{B_{c(i)}N}$.

The throughput maximization criterion results in the selection of a scheduled vector of users that achieves the largest sum rate among all possible vectors of users. Those users who have better channel gains have a higher likelihood to be selected by an MT scheduler. Thus, users with poorer channel gains may be very infrequently (and potentially never) selected by the scheduler, which is not fair. In PF scheduling, each user has a weight related to its priority for being chosen by the scheduler. The scheduler adjusts each weight based on the average achievable rates in the user’s history. A PF scheduler chooses those users whose instantaneous rates relative to their average rates are better than the others and uses a weighted sum rate as its scheduling metric, i.e., the combination of those users with maximum weighted sum rate will be chosen to be scheduled. If a user has been selected by the scheduler often, its weight for the next interval will be decreased (as its average rate increases), i.e., its chance to be chosen in the next scheduling interval diminishes. Meanwhile, another user with a worse channel matrix may have more opportunity to be scheduled in the next interval simply by having higher weight. Using this method provides more fairness in the network among all users.

In each cluster, the maximum achievable weighted sum rate *WSR*_*c*(*i*)_ is given by^3^

6 $$ {\begin{aligned} &\qquad {WSR}_{c(i)} = \max_{\pi_{c(i)}^j:j \in \{1,2,\cdots,U_{c(i)}!\}} \\ &\max_{\left\{\mathbf{Q}_{c(i),\pi_{c(i),k}^{j}}\right\}_{k\in \{1,\cdots,U_{c(i)}\}} : \mathbf{Q}_{c(i),\pi_{c(i),k}^{j}}\succeq \mathbf{0}, \ \sum\limits_{\forall k} Tr(\mathbf{Q}_{c(i),\pi_{c(i),k}^{j}})\leq 1} \\ &\qquad\qquad\qquad \sum_{k=1}^{U_{c(i)}} \mu_{c(i),\pi_{c(i),k}^{j}}(t)R_{c(i),\pi_{c(i),k}^{j}}(t) \end{aligned}}  $$

where $\mu _{c(i),\pi _{c(i),k}^{j}}(t)$ is the priority weight of the *k*th user during the *t*th scheduling interval in cluster *c*(*i*). In PF scheduling, for the *l*th user out of *K*_*c*(*i*)_, $\mu _{c(i),l} (t) = 1/\bar {R}_{c(i),l} (t)$, where $\bar {R}_{c(i),l} (t)$ is the average achievable data rate of the *l*th user at time *t*, averaged over a window of the past *t*_*c*_ intervals. In each time interval, $\bar {R}_{c(i),l}(t)$ (and thus *μ*_*c*(*i*),*l*_(*t*)) is updated by an exponential filter as

7 $$ {}\bar{R}_{c(i),l} (t+1) = \left\{ \begin{array}{ccc} \left(1-\frac{1}{t_{c}}\right)\bar{R}_{c(i),l} (t) &\text{if the } l\text{th user is }\\ + \frac{R_{c(i),l}(t)}{t_{c}} \qquad &\text{scheduled in }\\ &\text{interval } {t},\\ \left(1-\frac{1}{t_{c}}\right)\bar{R}_{c(i),l} (t) &\text{otherwise} \end{array}\right.  $$

*R*_*c*(*i*),*l*_(*t*) is the instantaneous rate of the *l*th user, and is obtained from (5), assuming the *l*th user is scheduled in position *k* of the ordered scheduling vector $\pi _{c(i)}^{j}$. One important special case of achievable weighted sum rate maximization is MT, which is defined by setting *μ*_*c*(*i*),*l*_ to a constant of 1 for all users. Let us define the best ordered user vector as *π*^∗^. Then, in any clustering pattern *i*, the maximum average achievable weighted sum rate over the area of an arbitrary macrocell, averaged over time *t* when using pattern *i*, is given as

8 $$ \begin{aligned} {}\mathbb{E}_{t} \!\left(WSR (t,i) \right) \,=\, \mathbb{E}_{t} \!\left(\sum\limits_{k=1}^{U_{c(i)}} \mu_{c(i),\pi_{c(i),k}^{*}}(t)R_{c(i),\pi_{c(i),k}^{*}}(t)\!\right)\!/ w_{c(i)} \end{aligned}  $$

where *w*_*c*(*i*)_ is the number of macrocells in cluster *c*(*i*).

To solve the optimization problem in (6) using (5) as $R_{c(i),\pi _{c(i),k}^{j}}$, we must consider the $\mu _{c(i),\pi _{c(i),k}^{j}}(t)$ weights when calculating $\mathbf {Q}_{c(i),\pi _{c(i),k}^{j}}$ using the water-filling algorithm, which allocates power over the eigenmodes of the block-diagonal matrix formed using the effective channel matrices^4^$\mathbf {G}_{c(i),\pi _{c(i),k}^{j}} = \tilde {\mathbf {H}}_{c(i),\pi _{c(i),k}^{j}}\mathbf {V}_{k-1}^{0}$. The user selection within a cluster is performed by using a SAS algorithm similar to what we proposed in [35] and described in Algorithm 3 therein. For ease of reference, the pseudocode of the SAS algorithm is described in Algorithm 1 here. The main difference between [35] and here is that the solution values *s*_**x**_ and $s_{\hat {\mathbf {x}}}$ are now achievable weighted sum rates as per (6). The ordered user vectors **x** and $\hat {\mathbf {x}}$ are used for $\pi _{c(i)}^{j}$ in (6). The rest of the operation of the algorithm is unchanged. The SAS algorithm operates in parallel separately for each cluster.


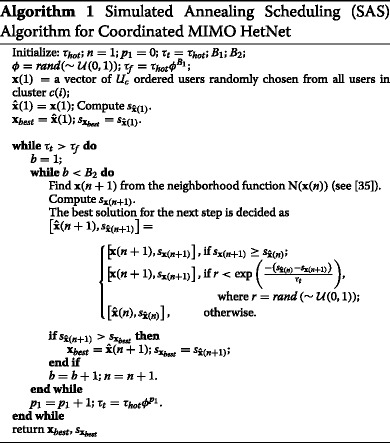


Two positive variables *B*_1_ and *B*_2_ limit the iterations and control how closely the algorithm approaches the optimal solution with the trade-off of the algorithm’s performance and its acceptable complexity. The larger the values of *B*_1_ and *B*_2_, the closer the algorithm comes to the optimal solution, but the computational complexity also increases as the algorithm iterates longer. The SAS algorithm starts with the variable *τ*_*t*_ (analogous to the temperature in metallurgical annealing) equal to *τ*_hot_. It continues until *τ*_*t*_ “cools” to the value of $\phantom {\dot {i}\!}\tau _{f} = \tau _{\text {hot}} \phi ^{B_{1}}$. *ϕ* is a uniformly distributed random variable in the interval (0,1). The neighborhood function N(**x**(*n*)) at random either deletes a random user from the vector **x**(*n*), adds a random unscheduled user (if possible), replaces one scheduled user with a random unscheduled one, or swaps the encoding order position of two random scheduled users. Each of these actions has an equal probability of being chosen.

We refer the reader to [35] for more details on our SAS algorithm. Note that no change to the scheduling algorithm is required for rotating clustering; better cluster patterns for users are automatically detected by the algorithm through the corresponding more favorable channel gains and/or achievable rates during that pattern, making the users more likely to be scheduled during those better patterns.

## Simulation setup and results

In this section, the simulations of the proposed rotating clustering mechanisms are presented and compared with fixed clustering of the otherwise identical cooperative HetNet employing the SZF-DPC precoding technique and SAS algorithm. Both MT and PF scheduling are considered. The average achievable sum rate and the average achievable rate per user are determined using the Monte Carlo simulation method. The numbers of transmitter and receiver antennas are assumed to be *N*_macro_=*N*_pico_=2 and *M*=2, respectively. These values enable a manageably low simulation complexity, yet still allow a demonstration of the effect of rotating clusters in coordinated MU-MIMO systems. It is assumed that the ISD in both layouts has the same value of^5^ *D*=1732 m. In the six-cell layout, there are 12 pico BSs spaced evenly on the imaginary circle with radius 693 m around each macro site. For the three-cell layout, four picos are located on a circle of radius 356 m centered at the center point of each macrocell coverage area^6^. The scaling factor for the SNR, i.e., *Γ*_0_ in (1), is set to result in an SNR of 9.6 dB at the distance *R*_*m*_=866 m in the six-cell layout, and an SNR of 4.7 dB at *R*_*m*_=1155 m for the three-cell layout. In both cases, *R*_*m*_ is measured from the macro site in the direction of the BS antenna boresight. The transmitting power *P*_*t*_(*b*) of each macro BS is 40 times greater than that of each pico BS. The path loss exponent *α*(*b*) is assumed to be 3.91 and 3.67, and the standard deviation of the log-normal shadow fading is *σ*_*ρ*_=6 and 4 dB, respectively, for macro and pico BSs^7^.

The total interference from the other clusters outside the target cluster is approximated by the complex Gaussian random vector with zero mean and standard deviation *σ*_*I*_. The value of *σ*_*I*_ is measured and averaged over the area of an entire cluster by employing similar methodology for both layouts as was used in [35]. The mean value of *σ*_*I*_ in the six-cell layout is measured as 13.8. In the three-cell layout, all five cluster patterns are not exactly symmetric nor statistically identical to each other. As depicted in Fig. 2, the cluster patterns in Fig. 2a, c, and e are similar in that for all three patterns, two sites contribute to a given cluster. Of the three macro BSs covering the cluster, two of them are co-located at the same site. However, in the cluster patterns of Fig. 2b, d, there are three equidistant macro BSs per cluster, with each BS belonging to a different site. This difference creates asymmetry in some features of these patterns. Most notably, the mean standard deviation of the interference across the clusters of Fig. 2a, c, and e is equal, but different from that of Fig. 2b, d, with values of 21.3 and 26.3, respectively, for the two cases. We account for these differences in our simulations. In the SAS algorithm, we use parameter values corresponding to SA-m (with memory) case #18 in [35]; we refer readers to that reference for details. A summary of the simulation parameters and their values is provided in Table 1.

**Table 1:** Simulation setup parameters and values for six-cell and three-cell layouts

	Six-cell layout	Three-cell layout
Number of Tx antennas per macro BS, *N*_macro_	2	2
Number of Tx antennas per pico BS, *N*_pico_	2	2
Number of Rx antennas per user, *M*	2	2
Macro antenna pattern	Directional [37];	Directional [37];
(including 3-dB beamwidth *θ*_3dB_)	*θ*_3dB_=35°	*θ*_3dB_=70°
Pico antenna pattern	Omnidirectional	Omnidirectional
Path loss exponent, *α*	3.91 (macro); 3.67 (pico) [32]	3.91 (macro); 3.67 (pico) [32]
Log-normal shadow fading standard deviation, *σ*_*ρ*_	6 dB (macro); 4 dB (pico) [32]	6 dB (macro); 4 dB (pico) [32]
Inter-site distance, *D*	1732 m	1732 m
Distance from pico BS to macro site	693 m	–
Distance from pico BS to center of macrocell coverage area	–	356 m
Reference distance, *R*_*m*_	866 m	1155 m
Macro SNR at distance of *R*_*m*_ along antenna boresight	9.6 dB	4.7 dB
Interference standard deviation, *σ*_*I*_	13.8	21.3 (patterns a, c, and e);
		26.3 (patterns b and d)
PF averaging window size, *t*_*c*_	100	40
SAS parameters	Case #18 in [35]:	Case #18 in [35]:
	*B*_1_=*B*_2_=50, $\phi = {rand}(\sim \mathcal {U}(0,1))$	*B*_1_=*B*_2_=50, $\phi = {rand}(\sim \mathcal {U}(0,1))$

### Simulation results for six-cell layout

**Fig. 3:**
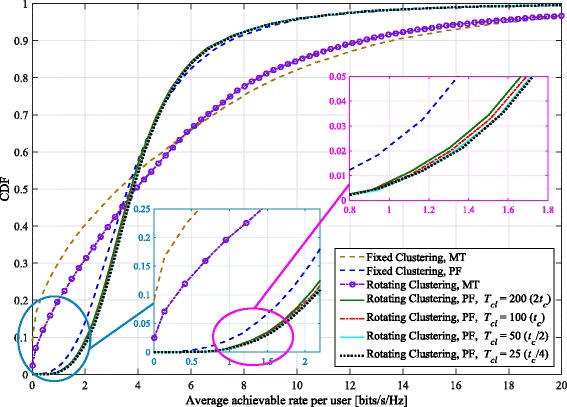
CDF of average achievable per-user rate with both proportionally fair (PF) and maximum throughput (MT) scheduling metrics, comparing proposed rotating clustering scheme and fixed clustering for six-cell layout, using simulated annealing scheduling and SZF-DPC precoding. *K*_*c*_=12, *N*_macro_=*N*_pico_=2, *M*=2

The clustering pattern is changed every *T*_*cl*_ scheduling intervals, and we set *t*_*c*_=100 for the size of the averaging window in the PF metric. We consider a minimum of four full sets of rotations through both patterns for our simulations (with that minimum occurring at the slowest rotation speed). The cumulative distribution functions (CDFs) of the average achievable rates per user for *K*_*c*_=12 and various *T*_*cl*_ values are depicted in Fig. 3. As this figure shows, rotating clustering has the most benefit in particular for those users with poorer channel gains (e.g., those with an average achievable rate around the 5th percentile). In comparison, users with better channels (who achieve higher average rates, such as those around the 90th percentile) see their average achievable rate drop with rotating clustering. The poorer 5th percentile users are those having low SINRs in a cluster in one of the patterns. Their achievable rates significantly benefit from rotation because they are located in a position in the cluster where they can have a better SINR after the rotation. The better users trade off their average achievable rate to provide higher overall fairness to the system.

**Fig. 4:**
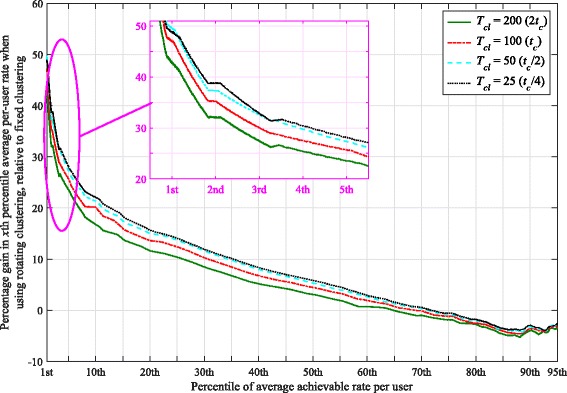
Comparison of percentage gain in the *x*th percentile average achievable rate per user of the rotating clustering scheme relative to fixed clustering, for different rotation speeds and using proportionally fair (PF) scheduling in the six-cell layout. Other parameters are the same as in Fig. 3

Considering different rotation speeds, Fig. 3 demonstrates that the rotation rate has an impact on each user’s average rate. This is the most easily seen for lower-rate users like those around the 5th percentile. The 5th percentile per-user rates generally increase with decreasing *T*_*cl*_, but eventually reach an upper limit, where even faster rotation yields no further significant gains. With PF scheduling, the gain in 5th percentile rate over that achieved with fixed clustering ranges between 23.5% to 28%. The gains in rates per user with PF scheduling are further emphasized in Fig. 4, which depicts, for each percentile of the average per-user rate, the percentage gain in average achievable per-user rates obtained when using rotating clustering, relative to the rates achieved with fixed clustering. As can be seen, the majority of the achieved per-user rates (those up to about the 65th to 72nd percentile) experience a gain with rotating clustering, at the expense of the rest. However, even at those remaining upper percentiles, the relative loss in rate is much smaller than the relative gain for the lower percentiles; at worst, the upper percentile rates drop by about 5%.

In each cluster, there are some users with particularly poor channel gains and even cluster rotation cannot help improve them much (such as those users located near the cell borders). These users have a very low chance to be selected by the MT scheduler; their average achieved rate is very close to (and sometimes equal to) zero, especially for fixed clustering. Cluster rotation helps these users by occasionally giving them better channels; however, there still remain some users that suffer from starvation. Nonetheless, although the probability of starvation is still non-zero for MT scheduling, it is reduced significantly (by a factor of over half for *K*_*c*_=12) by using cluster rotation, as seen in Fig. 3.

With rotation, the best users’ average achievable rates are reduced because they may be scheduled less often; however, they achieve higher instantaneous rates when they are selected. As an example, consider a theoretical extreme case of maximum throughput scheduling, where with fixed clustering, a few users would be scheduled most of the time with high throughput. With rotating clustering, instead about twice as many users would be scheduled overall, half of this total in each clustering pattern. As they would be scheduled about half as often (mostly only during their favorable pattern), their average achievable rate would also be about halved. However, they would achieve somewhat better instantaneous rates when scheduled, leading to an increased (albeit slightly) achievable sum rate. Similar and larger effects on the instantaneous and average achievable rates are seen with PF scheduling. As expected, while the average sum rate of PF is less than that of maximum throughput scheduling, its 5th percentile average achievable per-user rates are higher than for maximum throughput, and there is less overall variation in the average per-user rates achieved.

**Fig. 5:**
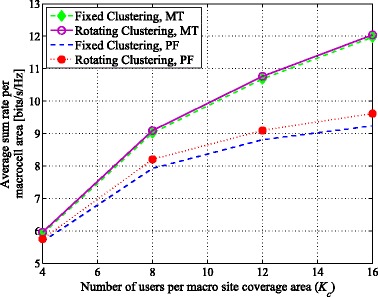
Average achievable sum rate vs. *K*_*c*_ with maximum throughput (MT) and proportionally fair (PF) scheduling metrics over the area of a macrocell in an arbitrary cluster, comparing proposed rotating clustering scheme and fixed clustering for six-cell layout, using simulated annealing scheduling and SZF-DPC precoding. *N*_macro_=*N*_pico_=2, *M*=2, *T*_*cl*_=*t*_*c*_=100

Figure 5 shows the average achievable sum rate for maximum throughput and proportionally fair scheduling vs. *K*_*c*_ over the area of a macrocell in an arbitrary cluster. In Fig. 5, *T*_*cl*_=100, which is equal to *t*_*c*_. As seen, rotating clustering outperforms fixed clustering for both scheduling metrics. However, rotating clustering increases the average achievable sum rate with PF relatively more than with maximum throughput. For instance, for MT scheduling, rotating clustering provides slightly higher throughput, increasing about 0.85% and 0.75% for *K*_*c*_=4 and *K*_*c*_=12, respectively, while for PF scheduling, the throughput is more significantly higher (about 1.4% and 3.2%, respectively, for *K*_*c*_=4 and 12). This is expected because the users that take the most advantage of the rotation are the users either near the border of the cluster or in poor coverage areas, who are scheduled more often with PF than with maximum throughput.

**Fig. 6:**
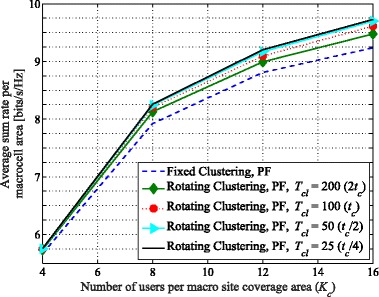
Average achievable sum rate vs. *K*_*c*_ over the area of a macrocell in an arbitrary cluster, comparing various *T*_*cl*_ values for proposed rotating clustering scheme for six-cell layout, using simulated annealing scheduling with proportionally fair (PF) scheduling metric (*t*_*c*_=100) and SZF-DPC precoding; *N*_macro_=*N*_pico_=2, *M*=2

In Fig. 6, the average achievable sum rate vs. *K*_*c*_ for proportionally fair scheduling considering different rotation speeds is presented. As seen, while rotating clustering still outperforms fixed clustering in terms of sum rate, faster rotation (i.e., smaller *T*_*cl*_) yields diminishing gains. For instance, rotating clustering provides higher throughput with respect to fixed clustering with faster rotation^8^, increasing by about 2.1%, 3.2%, 4.1%, and 4.5% with *K*_*c*_=12 for *T*_*cl*_=200, 100, 50, and 25, respectively. Considering an upper limit seen in the sum rate, rotating faster would not provide any further significant increase, but would increase complexity in signaling overhead for cluster setup. For our system, the limit is reached at about *T*_*cl*_=50 (half of *t*_*c*_). Smaller values of *T*_*cl*_ (e.g., *T*_*cl*_=25) yield almost no additional sum rate; the additional gain in sum rate relative to fixed clustering is only a few tenths of a percentage.

Assuming the scheduler has selected a user with poor channel gains in a cluster, the chance of this user being chosen again by the scheduler is comparatively low since its priority weight will have likely dropped in accordance with the update of its average rate by the exponential filter. The user’s priority will gradually increase as time passes if the user is not scheduled. If the cluster pattern change interval is smaller than *t*_*c*_, the possibility of that user being in a better position in another cluster pattern, and consequently dramatically improving its priority by virtue of its higher SINR and thus achievable rate, is increased. Similarly, a user with high SINR in a cluster that finds itself near a cluster edge after the pattern rotates will have its priority suddenly drop. It will be less likely to be scheduled until either the PF scheduling window passes or the cluster pattern rotates back, whichever comes first. Hence, faster rotations relative to *t*_*c*_ can potentially result in higher priority weights and higher sum rates. However, decreasing *T*_*cl*_ does not imply a linear increase in priority weights.

The upper limit on the performance with increasing rotation speed is understandable. In the PF scheduler, there are two factors influencing whether a user is scheduled: its potential instantaneous rate and its weight. The former is largely influenced by the cluster rotation, whereas the latter, among other things, is an indicator of how long the user has gone without being scheduled. The weight is updated with an exponential filter, meaning its value increases exponentially with time. Specifically, the increase is determined relative to the time constant (averaging window) *t*_*c*_, expressed as a number of scheduling intervals. If a user is not scheduled for *ν* scheduling intervals, its weight will increase by a factor of roughly exp(*ν*/*t*_*c*_). If the clustering pattern rotation interval is small relative to *t*_*c*_, the conditions for a user will become favorable or unfavorable sooner than the time it takes for the weight to change significantly. In other words, when *T*_*cl*_ is a fraction of *t*_*c*_, the weights change little between the instances in which a given user is scheduled. Hence, the specific value of *T*_*cl*_ is no longer much of a factor, leading to the upper limit on (saturation of) the performance with increasing rotation speed.

With larger *K*_*c*_, cluster rotation is more effective at increasing the sum rate; for example, the achievable gains with *T*_*cl*_=100 are 1.4% and 3.2% for *K*_*c*_=4 and 12, respectively, using PF scheduling. Faster rotation with larger *K*_*c*_ is more beneficial. For instance, with *K*_*c*_=8 and comparing the rotation speed of *T*_*cl*_=200 to *T*_*cl*_=100 and 50, the sum rate gains are 0.9% and 1.4%, respectively, whereas with *K*_*c*_=16, the gain increments are 1.4% and 2.4%. The probability of having users that are located in the cluster with favorable channel gains is increased with larger *K*_*c*_, simply as a result of multiuser diversity. Thus, being able to schedule more of these users improves the sum rate. Recall, though, that users are uniformly distributed over the entire coverage area. This statistically results in a higher proportion of users who are farther from a BS (with poorer channel gains) than those who are nearer. An increase in *K*_*c*_ thus also means an increase in the total number of “poorer” users. As was seen earlier, it is those users who benefit the most from rotation, explaining further why the gains with rotation are better with higher *K*_*c*_.

An increase in *K*_*c*_ also means more delays in scheduling users, as a larger pool competes for the same limited resources. Faster rotation also means less of a wait for any given user’s cluster pattern to be at its best, and thus potentially for the user to be scheduled, improving its average rate. In other words, faster rotation somewhat compensates for the increased scheduling delays as *K*_*c*_ grows, thus leading to the higher gains in rate observed with faster rotation at larger *K*_*c*_.

**Fig. 7:**
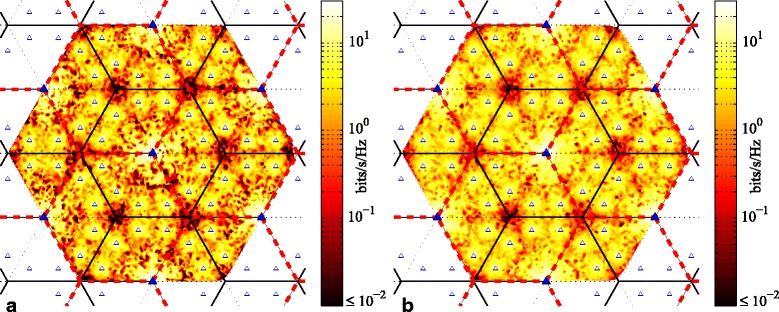
Average achievable rate per user based on user position within cellular network, with MT scheduling metric for six-cell layout, using simulated annealing scheduling and SZF-DPC precoding; *N*_macro_=*N*_pico_=2, *M*=2, *K*_*c*_=12. **a** Fixed clustering. **b** Rotating clustering (*T*_*cl*_=100)

**Fig. 8:**
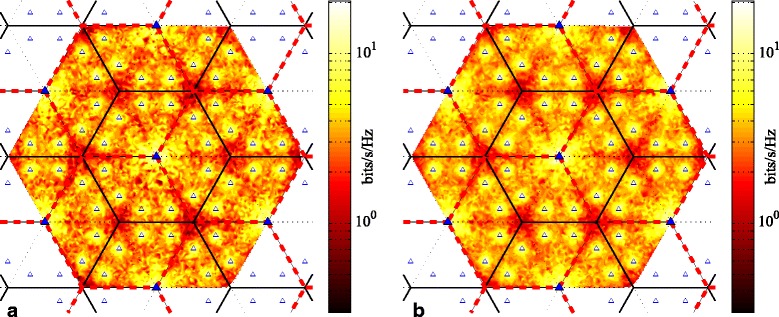
Average achievable rate per user based on user position within cellular network, with PF scheduling metric for six-cell layout, using simulated annealing scheduling and SZF-DPC precoding; *N*_macro_=*N*_pico_=2, *M*=2, *K*_*c*_=12. **a** Fixed clustering. **b** Rotating clustering (*T*_*cl*_=100)

Figures 7 and 8 further demonstrate the effects of rotating clustering (with *T*_*cl*_=100) by depicting the average achievable rate per user based on the user’s position within the cellular network; Fig. 7 depicts MT scheduling, whereas Fig. 8 depicts PF. Figures 7a and 8a show the average achievable rates with fixed clustering. The highest rates are unsurprisingly achieved by users nearest to a BS, especially macro BSs, but also to a lesser extent pico BSs. The lowest achievable rates (darkest red) are seen near any macrocell corner where three clusters meet. We will henceforth refer to these areas as the “corner” areas for shorthand. Lower rates are also seen in the area around the border of each cell, whereas somewhat higher achievable rates (orange to yellow to white) are seen near areas that correspond to the directions of the macro BS boresights. For any macro BS, users located near the left or right borders of the cell (i.e., at a 30° angle relative to the antenna boresight) receive weaker signals on account of the antenna directivity pattern. This situation is at its worst near the corners of the macrocells, at the furthest distance from the site. Furthermore, although the corners are surrounded by picos, the received signals from those picos are also weak due to being beyond the picocell borders. Hence, even with clustering (either fixed or rotating) and coordination, users in the corners achieve consistently smaller user rates, compared to the users in other locations of the cluster.

Figures 7b and 8b show the achievable rates with rotating clustering. In comparison, the average rates (or the colors) are more evenly distributed over the entire area, indicating higher overall fairness. As expected, the largest increase in average achievable rate is experienced by users that are closest to the cluster borders in the fixed scheme. Those users previously received the worst SINRs and/or the most ICI. In contrast, most users who are located close to the macro BSs or near the directions of the macro boresights receive very good signal power. The largest decrease in average achievable rates relative to the fixed scheme is by that latter group of users, as well as to a lesser extent users nearby pico BSs. It is these latter users who trade off some of their average rates to provide more fairness and uniformity of throughput across the coverage area. It is interesting to note that there are fairly large areas where the average achievable rate of a user does not change much with rotating clustering. These areas and users are those located in the interior of the cluster. Their conditions (in terms of both useful signals and interference) are more or less the same under either cluster pattern. Hence, rotation does not change their situation much, and so, their achievable rates do not change appreciably from the fixed scheme either. There is also little difference in the rates seen in the cell corner areas with rotation. The rather small changes that exist there are not readily visible in Figs. 7 and 8. However, where three clusters meet in the fixed scheme, those corners do experience a small boost in rates with rotation due to periodically increased coordination. Likewise, the corners at the cluster centers in the fixed scheme see a small decrease in rates with rotation, as signals received there are no longer always coordinated from all nearby BSs.

### Simulation results for three-cell layout

Similarly to the previous section, the clustering pattern changes (rotates) every *T*_*cl*_ scheduling intervals. For the six-cell layout, as described in Section 3.1, we simulate for a minimum of four complete sets of rotations. Assuming *T*_*cl*_=200, which corresponds to the slowest (non-zero) rotation speed, the total number of simulated channel realizations (or scheduling intervals) for every drop of users is thus 1600. To compare the three-cell layout with the six-cell layout, we consider two different scenarios. First, we keep the same total number of 1600 channel realizations per drop with four complete sets of rotations between the five clustering patterns. We also maintain the longest pattern duration as 2*t*_*c*_. Thus, *t*_*c*_ for the PF window becomes 1600 realizations/drop ÷4 sets/drop ÷5 patterns/set ÷2 windows/pattern =40 (realizations per window). This gives us different rotation speeds corresponding to pattern durations of *T*_*cl*_=[10,20,40,80]. Secondly, we keep the value of *t*_*c*_ equal to what we use in Section 3.1, i.e., 100, and the same pattern durations, i.e., *T*_*cl*_=[25,50,100,200]. With still four full sets of rotations at minimum, this results in 4000 channel realizations per drop. The simulations with these two scenarios were run for *K*_*c*_=12, and the results were compared with each other. We observed that the results of both scenarios were very close to each other^9^. Thus, we present only the results for where the number of channel realizations per drop equals^10^ 1600 and *t*_*c*_=40.

**Fig. 9:**
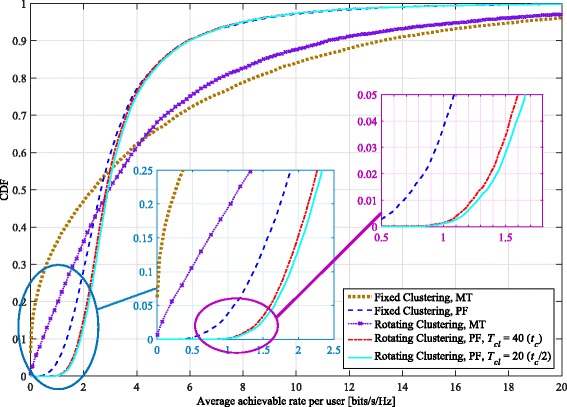
CDF of average achievable per-user rate with both proportionally fair (PF) and maximum throughput (MT) scheduling metrics, comparing proposed rotating clustering scheme and fixed clustering for three-cell layout, using simulated annealing scheduling and SZF-DPC precoding. *K*_*c*_=12, *N*_macro_=*N*_pico_=2, *M*=2

In Fig. 9, the CDF of the average achievable rates per user for *K*_*c*_= 12 is illustrated. Similarly to the results depicted by Fig. 3, rotating clustering again yields higher per-user rates for those who are located near cluster edges or in otherwise poor coverage areas in the three-cell layout (e.g., the users with rates around the 5th percentile). Comparison of these two figures is also interesting. As seen in Fig. 3 with MT scheduling, while rotating clustering improves the per-user rates in the six-cell layout, there are still some users whose achievable rates are very low, even to the point of starvation. However, cluster rotation in the three-cell layout improves the average throughput of users considerably such that the probability of very low rates and/or starvation becomes almost zero. There is a larger trade-off in the high-percentile users’ rates to achieve this, though.

In the three-cell layout and with PF scheduling, the rotation speed corresponding to *T*_*cl*_=*t*_*c*_ yields a per-user rate increase of about 0.5 bits/s/Hz at the 5th percentile, while the improvement with the six-cell layout is around two thirds of that. In relative terms, the 5th-percentile rate improvements with rotating clustering (at *T*_*cl*_=*t*_*c*_) over fixed in the six- and three-cell layouts are, respectively, about 25.7% and 47.3%. This indicates cluster rotation is even more effective at improving the cluster-edge user rates in the three-cell layout. Considering the faster rotation speed corresponding to *T*_*cl*_=*t*_*c*_/2, the additional 5th-percentile users’ rate increase (relative to *T*_*cl*_=*t*_*c*_) is about 1.3% in the six-cell layout, while this increase is about 3.7% using the three-cell layout. This shows that faster rotation in the three-cell layout is more beneficial than in the six-cell layout to help cluster-edge users to gain further higher throughput. This result is not surprising, considering the former rotates through a larger set of patterns.

**Fig. 10:**
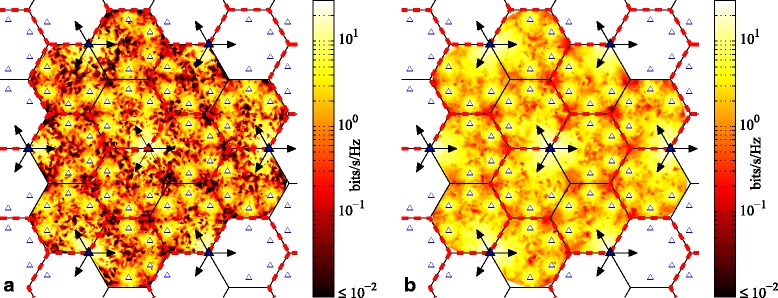
Average achievable rate per user based on user position within cellular network, with MT scheduling for three-cell layout, using simulated annealing scheduling and SZF-DPC precoding; *N*_macro_=*N*_pico_=2, *M*=2, *K*_*c*_=12. **a** Fixed clustering. **b** Rotating clustering (*T*_*cl*_=40)

**Fig. 11:**
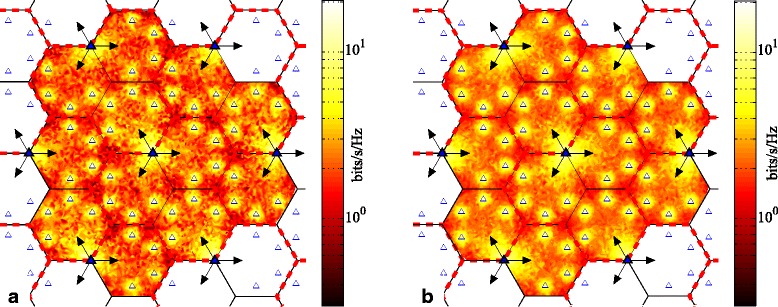
Average achievable rate per user based on user position within cellular network, with PF scheduling for three-cell layout, using simulated annealing scheduling and SZF-DPC precoding; *N*_macro_=*N*_pico_=2, *M*=2, *K*_*c*_=12. **a** Fixed clustering. **b** Rotating clustering (*T*_*cl*_=40)

We also investigate the user rates vs. their position within the network in Fig. 10 (MT scheduling) and Fig. 11 (PF scheduling). Basically, the influence of cluster rotation on achievable per-user rates with MT and PF scheduling is similar to what we have discussed in Section 3.1. As before, it is users located at the cell corners away from the sites that achieve the lowest rates. To a lesser extent, users near the middle of cells, not especially near a macro BS nor a pico BS, also achieve somewhat lower rates. As is obvious from Figs. 10 and 11, the achieved users’ rates are distributed more uniformly with rotating clustering than with fixed clustering for both MT and PF scheduling. Furthermore, the overall rates are increased. The achievable throughput of those users who are located in the corners of a cell is improved, and the size of the areas experiencing lower rates is diminished. This is particularly noticeable in Fig. 10 for MT scheduling; the colors in the cell corners increase from dark red to orange.

Cell-center users are also significantly affected by cluster rotation. Note that in fixed clustering, and in the patterns in Fig. 2a, c, and e, two parts of the cluster come from the same site. Because of the macro BS antenna pattern, the coverage of the beams from those two BSs has little overlap. Hence, those two cells are essentially impacted by, at best, two coordinated signals: one from their own cell and one from the third BS at the other site that contributes to the cluster. However, with rotation through the patterns in Fig. 2b, d, receiving a significant coordinated signal from three BSs is more common. Thus, users in those two cells in the cluster see the most improvement from rotation^11^. The trade-off now comes from users in the third cell of the fixed scheme, who with cluster rotation now are forced to experience less advantageous cluster patterns for the sake of overall network fairness.

**Fig. 12:**
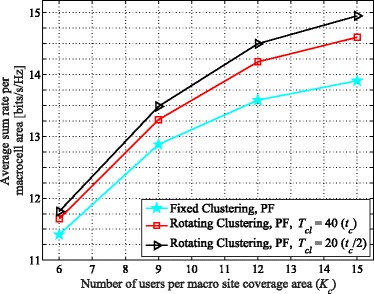
Average achievable sum rate vs. *K*_*c*_ with proportionally fair (PF) scheduling (*t*_*c*_=40) over the area of a macrocell in an arbitrary cluster, comparing various *T*_*cl*_ for proposed rotating clustering scheme and fixed clustering for three-cell layout, using simulated annealing scheduling and SZF-DPC precoding. *N*_macro_=*N*_pico_=2, *M*=2

Figure 12 demonstrates the achievable sum rates vs. *K*_*c*_ for the three-cell layout. Much like the six-cell layout, MT scheduling again does not display a gain in sum rate from cluster rotation, for similar reasons as described in Section 3.1. However, the throughput with PF scheduling does again increase considerably. For example, with *K*_*c*_= 12, comparing the achievable throughput of rotating vs. fixed clustering shows an increase of 4.5% and 6.7%, for *T*_*cl*_ equal to *t*_*c*_ and *t*_*c*_/2, respectively. Comparing the six- and three-cell layouts with the same number of users in each cluster, the six-cell layout generally yields higher values for area spectral efficiency on average. For example, the average total sum rates per macrocell for *K*_*c*_=12 are 8.8 and 13.6 bits/s/Hz, respectively, for the six- and three-cell layouts with PF using fixed clustering. Yet, also note that the macrocell area in the six-cell layout is half of that in the three-cell layout (see Endnote 5). Hence, the sum rate per unit area (e.g., per km^2^) is larger in the six-cell layout. However, the per-user rate improvement achieved with cluster rotation in the three-cell layout is much more significant. Faster rotation yields higher gains in this layout than in the six-cell layout. For instance, the additional sum rate improvement achieved by setting *T*_*cl*_=*t*_*c*_/2 relative to *T*_*cl*_=*t*_*c*_ is 2.1% in the three-cell layout, while this gain in the six-cell layout is only 0.8%. Although faster rotation improves the average sum rate, the upper limit for higher speeds of rotation is still similar to what we described in Section 3.1. Thus, we only present the results for *T*_*cl*_ equal to *t*_*c*_ and *t*_*c*_/2. All these comparisons demonstrate that the rotating cluster method overall helps the system performance in the clover-leaf-shaped network layout more than in the hexagonal-shaped layout.

### Comparison of rotating cluster method with dynamic cluster method

In [30], a dynamic clustering method was proposed and compared with static clustering. There are several differences in the simulation methodology in that paper compared to our own (e.g., a single-tier homogeneous network, differing numbers of users and antennas, the precoding method). However, it can still serve as a rough guide for the performance benefits of a fully dynamic scheme over static clustering and that can be used for comparison with our less complex rotating clustering method. The simulations in [30] assumed three macrocells per site, four transmit antennas per macrocell, two antennas per user, and PF scheduling. The dynamic scheme was shown to have approximately an 18% gain in average sum rate per cell (Fig. 5 in [30]) vs. a static clustering scheme of three cells per cluster with one macrocell contributing from each site (similar to the macrocells in our three-cell layout). The gain in 5th percentile user rate was about 22% (Fig. 6 in [30]); this gain approximately doubled when the maximum cluster size of the dynamic scheme increased from three to six, though at the same time also enforcing that just a single data stream be sent to each user (Fig. 9 in [30]).

In comparison, for our six-cell layout, the 5th percentile user rates for *K*_*c*_= 12 and PF scheduling increase by about 23.5% to 28% over static clustering for the minimum and maximum examined rotation speeds. In the three-cell layout, the gains were even higher, i.e., 47–53% for *K*_*c*_= 12. The gain in sum rate per cell was lower though, about 2.1–4.5% for *K*_*c*_= 12 in the six-cell layout, and about 4.5–6.7% for *K*_*c*_= 12 in the three-cell layout. However, we remind the reader that our rotating cluster scheme was primarily designed to help cluster-edge users (e.g., those around the 5th percentile); any gains in cell sum rate are an added secondary bonus. Hence, we can see that, compared to the fully dynamic scheme of [30], our rotating scheme provides gains for cluster-edge users that are on par with or better than those in [30], but at the trade-off of lower gains in the sum rate per cell.

## Conclusions

We have considered the downlink of a coordinated heterogeneous MIMO cellular network (including macro and pico BSs) in hexagonal-shaped and clover-leaf-shaped cell layouts. A different rotating set of cell-cluster patterns has been proposed for each layout. The proposed scheme’s performance, considering two user scheduling metrics, has been evaluated by simulation. The results demonstrate that the proposed cluster rotation scheme performs significantly better than fixed clustering for both metrics and both layouts, while being less complex than fully dynamic clustering. The average achievable rate per user has been improved for cluster-edge users, which is the primary goal of cluster rotation. Additionally, the average achievable sum rate has also been improved, which is a secondary benefit. User rates also become more evenly distributed over the network coverage area. A comparison with fully dynamic clustering shows cluster rotation yields similar performance gains for cluster-edge users.

We have also evaluated the effect of different cluster rotation speeds on the system performance in both cellular layouts. The results demonstrate improving performance with increasing speed of rotation up to an upper limit on that speed, beyond which further increases yield no notable additional gains in sum rate or per-user rate. The clover-leaf-shaped layout gains more in performance from cluster rotation than the hexagonal-shaped one. This indicates in general that some network layouts (and their associated cluster patterns) may benefit more from cluster rotation than others.

## Abbreviations

BS: Base station
CDF: Cumulative distribution function
CoMP: Coordinated multipoint
CSI: Channel state information
DPC: Dirty paper coding
HetNet: Heterogeneous network
ICI: Inter-cluster interference
i.i.d.: Independent and identically distributed
IMT: International Mobile Telecommunications
ISD: Inter-site distance
ITU-R: International Telecommunication Union – Radiocommunication Sector
LTE: Long Term Evolution
MIMO: Multiple-input multiple-output
MT: Maximum throughput
MU-MIMO: Multiuser multiple-input multiple-output
NLoS: Non-line-of-sight
PF: Proportionally fair
SAS: Simulated annealing (user) scheduling
SINR: Signal-to-interference-plus-noise ratio
SNR: Signal-to-noise ratio
SZF-DPC: Successive zero-forcing dirty paper coding
UMa: Urban Macro
UMi: Urban Micro
WSR: Weighted sum rate

## Footnotes

1 This terminology differs from what we used in [19, 20]. In our earlier papers, we referred to each of the six parts of the hexagonal area as a sector, and the six sectors together constituted a single cell. Now, we instead refer to the former sectors as cells, and the six macrocells have their BSs co-located at one site.

2
*R*_*m*_ is defined as the farthest distance from the macro BS to the edge of its cell in the direction of its boresight. Since we fix the value *D* of the ISD for both layouts in our simulations, *R*_*m*_ in the six-cell layout is *D*/2 while it is 2*D*/3 for the three-cell layout.

3 In (6), the sum-trace (transmit power) constraint of 1 on $\mathbf {Q}_{c(i),\pi _{c(i),k}^{j}}$ assumes the transmit power *P*_*t*_(*b*) for BS *b* is embedded in the channel matrix, as we have done in (1); this method is equivalent to not embedding the power in the channel matrix, but instead using a transmit power constraint of *P*_*t*_(*b*) for BS *b*. Embedding the power in the channel matrix also allows the rates to be calculated independently of the type of transmission nodes (and their specific transmitted power levels) used in the network. For simplicity of calculation, we have used a sum-power constraint over all coordinated BS antennas instead of per-BS constraints.

4 This is similar to [14, Eq. (32)], except replacing the |**h**_*k*_**w**_*k*_|^2^ terms there with squared singular values of $\mathbf {G}_{c(i),\pi _{c(i),k}^{j}}$.

5 Using this ISD, the circle circumscribing the macro site coverage area in the six-cell layout has a radius of $D/ \sqrt {3}$, which equals 1 km. The radius of the circle circumscribing a single macrocell in the three-cell layout is *D*/3. Using simple geometry, the area covered by a macrocell in the three-cell layout can be calculated as twice to that of a macrocell in the six-cell layout. Since the six-cell layout has twice as many macrocells, the total coverage area of a macro site is the same in both layouts.

6 In both layouts, the location of a pico BS is determined such that the border of its coverage area touches the border of the macrocell coverage area it is overlaid on. The picocell borders themselves are determined by where there is an equal SINR from the pico BS and from the macro BS of the macrocell that the picocell overlays. (The hexagonal picocell borders shown in the figures are an approximation to the true shape of the borders, meant to aid the visualization of the layout.)

7 The values of the path loss exponents and the shadow fading standard deviations used here for macrocells and picocells correspond to the Urban Macro (UMa) non-line-of-sight (NLoS) scenario and the Urban Micro (UMi) NLoS scenario, respectively, as found in [32].

8 A similar evaluation has been performed for MT; the results show no notable improvements with higher rotation speeds. This is also expected because MT overall gains very little in sum rate by using cluster rotation in contrast to PF.

9 This is likely as a result of the assumption that there is no temporal correlation in the small-scale fading component of the users’ channel gains, i.e., each realization of *z*_*c*(*i*),*k*_(*b*,*m*,*n*) in (1) is independent from any other. If there was temporal correlation, the results of the two scenarios would likely differ.

10 For certainty, we have simulated all cases for both scenarios and in each case the achieved results were similar. Aside from the fact that fewer realizations means less computation time for the simulations, using 1600 realizations for both the six-cell and three-cell layouts means a comparison of the results between the two layouts is somewhat more statistically equitable.

11 This is despite the clusters of the patterns in Fig. 2b,d experiencing more surrounding interference, as seen by their larger value of *σ*_*I*_ in the simulation setup.
